# Oral Hyaluronic Acid in Osteoarthritis and Low Back Pain: A Systematic Review

**DOI:** 10.31138/mjr.240724.oha

**Published:** 2024-12-31

**Authors:** Jozélio Freire de Carvalho, Josy Davidson

**Affiliations:** 1 Núcleo de Pesquisa em Doenças Crônicas não Transmissíveis (NUPEC), School of Nutrition from the Federal University of Bahia, Salvador, Bahia, Brazil; 2 Centro Universitário São Camilo, São Paulo-SP, Brazil

**Keywords:** hyaluronic acid, oral hyaluronic acid, osteoarthritis, rheumatological diseases, low back pain

## Abstract

**Background:**

Hyaluronic acid (HA) has been largely used in clinical practice for rheumatic diseases. However, the effects of oral HA on these diseases are poorly understood.

**Aim:**

To review articles evaluating oral HA’s effects on rheumatic patients.

**Methods:**

PubMed was searched for articles on oral HA and rheumatic diseases between 1966 and May 2024.

**Results:**

Eleven articles were found with 597 patients. The diseases investigated were OA (n=10) and low back pain (n=1). Age varied from 40 to 70 years old, and female gender ranged from 43% to 75%. Follow-up ranged from 4 weeks to 12 months. The oral HA dosage varied from 30 mg to 300 mg/day. Concerning outcome, 9/11 articles observed improvement in rheumatic diseases in the following parameters: VAS pain, WOMAC, joint function, SF-36, Lequesne index, and stiffness. Two studies evaluated cytokines and observed a reduction of them after oral HA therapy. Adverse effects were rare and mild.

**Conclusion:**

Oral HA seems to be a safe and effective therapy for OA and low back pain patients, although more studies should be done on the latter condition.

## INTRODUCTION

Osteoarthritis (OA) is a prevalent global condition that becomes more common with age and factors like obesity. Given the rise in these contributing factors, the frequency of OA is anticipated to increase in the coming years and decades. According to Safari et al.’s Global Burden of Disease analysis in 2017,^1^ OA is widespread across 195 countries, with an estimated 303.1 million hip and knee OA cases worldwide. The data also indicates an age-standardised increase in prevalence, incidence, and years lived with disability of approximately 8–10% since 1990.

Osteoarthritis (OA) is managed through non-pharmacological approaches such as physical therapy, exercise, insoles, weight management, and pharmacological interventions like analgesics and non-steroidal anti-inflammatory drugs. Nutraceuticals play a role in OA treatment, with substances like glucosamine, chondroitin, collagen, MSM, and Boswellia serrata extracts yield varying degrees of effectiveness.^2^ In addition, minimally invasive procedures like intraarticular glucocorticoid injections, prolotherapy, platelet-rich plasma therapy, and hyaluronic acid (HA) injections have significantly improved over placebo treatments.

Administering intraarticular injections carries risks such as bleeding, infections, and potential harm to the joints’ internal structures. While these injections have shown efficacy and safety, some patients may hesitate due to discomfort from multiple injections and the need for frequent clinic visits when using techniques like high molecular hyaluronic acid injections.

In this line,^3^ HA has been developed in an oral presentation to overcome the mentioned limitations. An oral administration of low molecular weight HA, which can be absorbed in the gastrointestinal tract, is a possible alternative therapy for these patients.^4^ In fact, studies have demonstrated the efficacy of oral HA in clinical practice, although with controversial results.

Therefore, this study’s objective was to review articles on the use of oral hyaluronic acid (OHA) in the treatment of rheumatic diseases.

## METHODS

### Literature review

A systematic literature screening of articles published in PubMed, EMBASE, and Scielo from 1966 to May 2024. We used the following MeSH entry terms: “oral hyaluronic acid” AND “rheumatic” OR “rheumatologic” or “low back pain” OR “osteoarthritis” OR “ gout” OR “fibromyalgia” were used. The literature search did not use language restrictions. We also screened all references for selected articles to identify additional publications. Exclusion criteria were review articles, in vivo and in vitro studies, and articles published in the grey literature.

Two authors (JFC and JD) initially performed the literature screening and independently selected the abstracts of the articles. Then, the authors independently read all selected full-text articles in the second part, following the PRISMA guidelines.^5^ Finally, a form was constructed to extract the relevant information from the manuscripts, such as authors, year of publication, number of patients studied, age, gender, disease duration, study follow-up, HA posology, control, outcomes, and side effects.

## RESULTS

**Table 1** summarises the studies of OHA treatment in rheumatic diseases.^6–15^ Eleven articles were found, including 597 patients.^6–16^ The countries that reported those selected articles were Italy (n=4), followed by the United States (n=3), Japan (n=1), and Taiwan (n=1). The diseases investigated were OA (n=10) and low back pain (n=1). Knee OA was studied in 9/11 articles, and knee and hip OA in 1/11.^6^ Regarding the study design, most were randomised double-blinded placebo-controlled trials (n=6), followed by randomised controlled trials (n=4) and double-blinded placebo-controlled trials (n=1).

**Table 1.** Summary of the studies on oral hyaluronic acid in rheumatic diseases.

**Author, year**	**Study design**	**Country**	**N, female sex**	**Age, years old**	**Disease**	**Follow-up study**	**HA dosage/day**	**Comparator**	**Outcome**	**Side effects**
Minoretti et al., 2024^16^	Randomised, single-blind trial		51, 68%	56.5 ± 4.3	Knee osteoarthritis	4 weeks	300mg of Syalox® 300 Plus	Cartijoint® Forte (glucosamine, chondroitin and curcuminoids) group and no treatment group	HA was better in reduce pain at rest, pain during movement, and on the WOMAC pain Subscale	3/17 OHA vs. 3/17 control. All mild (dyspepsia, flatulence and puritus)
Hill et al., 2023^6^	Randomised, double-blind, placebo controlled trial	United States and Korea	100, 57% Final: 75, 58.67%	58.0 ± 11.06	Knee and hip osteoarthritis	12 weeks	30mg of FlexPro MD®, one capsule once daily for 12 weeks	Placebo, one capsule once daily for 12 weeks	HA improved Korean-VAS joint pain score, Korean- WOMAC, and joint function scoring	Placebo 16% vs. HA 4%, p = 0.0455
Wang et al., 2021^7^	Randomised, double-blind, placebo-controlled study.	Taiwan	47, 75%	61.5 ± 9.9	Knee osteoarthritis	8 weeks	50 mg plus 750mg glucosamine, and 250mg chondroitin, a bottle in the morning under fasting condition once daily, 8 weeks	Placebo, a bottle in the morning under fasting condition once daily, 8 weeks.	HA reduced WOMAC pain, stiffness, physical function, and total score. HA improved SF-36 physical functioning and bodily pain domains SF-36 total score did not differ	ND
Wang et al., 2021^8^	Randomised, double-blind, placebo-controlled study.	Taiwan	80, 72%	58.4 ± 10.7	Knee osteoarthritis	8 weeks	50 mg plus 750mg glucosamine, and 250mg chondroitin, a bottle in the morning under fasting condition once daily, 8 weeks	Placebo, a bottle in the morning under fasting condition once daily, 8 weeks.	No differences in WOM-AC, SF-36, PSQI, and KIOOS in both groups.	None
Oliveiro et al., 2020^9^	Randomised controlled trial	Italy	30, ND	64.4 ± 6.9	Knee osteoarthritis	4 weeks	Artrocentesis + 120 mg of Ialoral® 1day, 4weeks	Arthrocentesis	HA improved WOMAC, Lequesne, and VAS pain. HA reduced IL-6, IL-8 and IL-10 in synovial fluid	ND
Ricci et al., 2016^10^	Randomised controlled trial	Italy	60, 47%	40–70	Knee osteoarthritis	12 weeks	300mg of Syalox 300 Plus®, 1 capsule/day for 20 days and hen 150mg of Syalox 300 Plus® 1 capsule/day for 20 days	Intra-articular HA 1.6% weekly for 3 weeks.	Both groups improved AKSS and VAS pain.	None
Panuccio et al. 2015^11^	Randomised, double-blind, placebo-controlled clinical trial	Italy	100, 47%	NA	Knee osteoarthritis	12 weeks	Intra-articular HA followed by a oral Compound^1^ 1 capsule/day, 3 months.	Intra-articular HA followed oral placebo, 1 capsule/day, 3 months.	No differences, although a trend to be better in VAS, KIOOS, Lequesne and TLKSS in OHA	GI discomfort (1) intra-articular HA +oral placebo group.
Nelson et al., 2015^12^	Double-blind, randomised, placebo-controlled study	United States	40, 43%	60 (51–75)	Knee osteoarthritis	12 weeks	80 mg of Oralvisc®, 1 capsule/day, 3 months	Placebo, 1 capsule/day, 3 months	HA improved pain, WOMAC pain. HA reduced leptin, bradykinin, IL-1 α, IL-1β, IL-6, IL-8, IL-12, IL-17α in serum and synovial fluid.	ND
Tashiro et al., 2012^13^	Double-blind, Placebo-Controlled Study	Japan	60, 77%	69.0 ± 1.0	Knee osteoarthritis	12 months	200mg, 4 hard capsules/day, 12 months	Placebo, 4 hard capsules/day, 12 months	Both groups improved JKOM. HA improved JKOM in patients < 70 years.	Gastric discomfort (2), reflux esophagitis (1), and appetite loss (1) HA group vs. skin irritation (2) in placebo
Kalman et al., 2008^14^	Randomised double-blind placebo-controlled trial	United States	20, 58%	57.7 ± 10.1	Knee osteoarthritis	8 weeks	80 mg, 1 capsule/day, 8 weeks	Placebo, 1 capsule/day, 8 weeks	Both groups had improvements in WOMAC pain, stiffness, physical function subscales, and in the aggregate score, but the magnitude of changes was higher in the HA group for WOMAC physical function and total symptoms. Quality of life improved in both groups.	1 HA vs. 2 placebo group
Fari et al., 2022^15^	Randomised controlled trial	Italy	60, 63%	40–80 Mean: 61	Low back pain	9 weeks	Fortigel collagen peptides 5000mg, Vitamin C 80mg, sodium hyaluronate 50mg, manganese 1mg, and cop-per 0.5mg for 20 days plus 9 sessions kine-sitherapy, 3x/week, 3 weeks	Kinesitherapy 3x/week, 3 weeks	Both groups improved after 3 weeks of treatment. Control group worsened VAS, ODI, and SF-12 after 6 weeks without treatment.	ND

ALSS: American Knee Society Score; IL: interleukin; NA: not available; ND: not described; VAS: visual analogic scale; WOMAC: Western Ontario and McMaster Universities Osteoarthritis Index; SF-12: 12-item Short Form Survey ; SF-36: 36-item Short Form Survey; PSQI: Pittsburgh Sleep Quality Index; KIOOS: Knee Injury and Osteoarthritis Outcome Score; Oralvisc®: HA (70 %) and Glycosaminoglycans; NF: not described; Ialoral®: 120 mg HA, 240 mg chondroitin sulphate, 300 mg bioactive oligopeptides of natural hydrolysed keratin, manganese and piperine; Syalox 300 Plus®: hyaluronic acid 300 mg; ? Boswellia serrata extract 100 mg.; JKOM: Japanese Knee Osteoarthritis Measure: Fortigel®: collagen peptide; ODI: Oswestry Disability Index; Compound1: hydrolysed low molecular weight collagen matrix providing high content of depolymerised HA and chondroitine sulfate, with methylsulfonylmethane, manganese and a milk glycoprotein; TLKSS: Tegner Lysholm Knee Scoring Scale; FlexProMD®: Antarctic krill oil (321 mg, astaxanthin (2 mg), and HA (30 mg).; Syalox® 300 Plus: 300 mg of HA and 100 mg of B. serrata.

Age varied from 40 to 70 years old, and female gender ranged from 43% to 75% in the included articles. Follow-up ranged from 4 weeks to 12 months. The OHA dosage varied from 30 mg to 300 mg/day. Two out of 11 trials received isolated OHA, and 9/11 received it combined with other substances, including glucosamine and chondroitin (2/9), collagen, methylsulfonylmethane, and chondroitin (1/9), ***B. serratia*** (2/9), glycosaminoglycan (1/9), chondroitin and keratin (1/9), and krill and astaxanthin (1/9). When available, the dosages of these other compounds were low. Seven out of 11 trials compared OHA with placebo; the other 4 studies compared with intraarticular HA 1/4, arthrocentesis 1/4, and kinesio-therapy 1/4, and 1/4 with glucosamine and chondroitine. Concerning outcome, 9/11 articles observed improvement in rheumatic diseases. In 2/11, no effect was observed. The effects were: pain reduction (4 of 9 studies), Western Ontario and McMaster Universities Arthritis Index (WOMAC) in 5/9, joint function in 2/9, SF-36 in 1/9, Lequesne index of severity for osteoarthritis in 1/9, and stiffness in 1/9. In 2/9 studies, improvements in the OHA group and the placebo were similar after treatment, although only OHA was significant for the most severe disease or younger patients below 70 years old.^13^

Two studies evaluated cytokines and observed their reductions after OHA therapy, including IL-1, IL-6, IL-8, IL-12, IL-17, and bradykinins in serum^12^ and l6, IL-8, and IL-10 in synovial fluid.^9^

Concerning adverse effects, they were seen in 2/11 in the OHA group and were mild; the events were more frequent in the placebo group than in the OHA group in 2/10, absent in 2/10, and the authors did not refer to them in 3/10 articles.

## DISCUSSION

This article is the first review of the therapeutic effects of oral HA supplementation in rheumatological disorders. The oral bioavailability of HA has been previously reported.^17^ The mechanism of the effects of oral HA may be due to its impact on intestinal Toll-like receptors.^18^

Hyaluronic acid is a large molecule in cartilage and synovial fluid. It comprises alternating N-acetyl-D-glucosamine and D-glucuronic acid units.^19^ With a molecular weight ranging from 6,500 to 10,900 kDa, HA serves various functions, such as lubrication, scavenging free radicals, and regulating cellular processes, including protein interactions. Its mechanism of action involves alleviating pain by enhancing the synthesis of extracellular matrix proteins, suppressing inflammatory mediators to prevent cartilage breakdown, reducing lymphocyte movement, and maintaining cartilage integrity in terms of thickness, area, and surface texture.

Regarding low back pain and HA, a recent systematic review included 18 studies with 1,496 patients with chronic low back pain caused by facet joint syndrome. The authors found the efficacy of the HA injection was similar to that of glucocorticoid injection.^21^ Furthermore, a study that prospectively evaluated HA in 10 patients with lumbar foraminal stenosis and used a single epidural injection of HA showed improvement in the straight leg raise test after the procedure.^22^ In summary, there are several possibilities for using HA in lumbar spine pathologies, including spine OA. The article we included in our review is the only one that used oral HA to treat low back pain with promising results.^15^

The present systematic review shows that almost all studies that evaluated OAH supplementation in OA and low back pain led to at least one benefit, with mild or absent adverse or toxic effects. Although, it is important to emphasise that 8 out of 10 trials herein studied, OHA was studied in combination with other substances, as methylsulfonylmethane, chondroitin, ***B. serratia***, glycosaminoglycan, keratin, krill, and astaxanthin.

The present study’s strengths are: 1. Only patients fulfilling the standardised criteria for rheumatological disorders were included, and 2. All kinds of study designs for using OHA in rheumatic diseases were included, except review articles, animal studies, and ***in vitro*** studies. In this way, the authors believe all published reports of OHA in rheumatic patients were captured.

Some limitations were seen in the studies herein evaluated. For instance, there were few participants in the study of low back pain. It is well known that this condition is universally present worldwide. OHA was largely used to treat knee OA in 8 out of 9 studies. Expanding the evaluation to other joints such as hands, spine, and hips would be interesting.

Future studies in this field should include more subjects with low back pain. In addition, it is desired to evaluate the effect of OAH in other rheumatological conditions, such as chondromalacia, tendonitis, greater trochanteric pain syndrome, and frozen shoulder, since injections of HA have produced good results in these conditions.^23–26^

## CONCLUSION

OHA seems to play a safe and efficacious role in treating osteoarthritis and low back pain. Eighty percent of the analysed studies demonstrated that OAH effectively reduces signs and symptoms of rheumatic conditions (pain, functionality, activity, and cytokine levels) with minor side effects. Based on the above, it is concluded that OAH surges are an alternative supplement to be explored in rheumatology, mainly in patients who do not want to be treated with intraarticular injections.
